# Effective long-term treatment with moss-produced factor H by overcoming the antibody response in a mouse model of C3G

**DOI:** 10.3389/fimmu.2025.1535547

**Published:** 2025-03-07

**Authors:** Todor Tschongov, Swagata Konwar, Jessika Kleindienst, Paulina Dabrowska-Schlepp, Andreas Busch, Andreas Schaaf, Christoph Schell, Manuel Rogg, Karsten Häffner

**Affiliations:** ^1^ Department of Internal Medicine IV, Medical Faculty, University of Freiburg, Freiburg, Germany; ^2^ Department of Biology, Albert-Ludwig University Freiburg, Freiburg, Germany; ^3^ Nonclinical Development, Eleva GmbH, Freiburg, Germany; ^4^ Institute for Surgical Pathology, Medical Center – University of Freiburg, Faculty of Medicine, University of Freiburg, Freiburg, Germany

**Keywords:** C3 glomerulopathy, complement system, protein replacement therapy, complement factor H, anti-drug antibodies, B-cell depletion, T-cell depletion

## Abstract

Complement-associated disorders are caused by the dysregulation and disbalance of the complement system, especially excessive activation. Most drugs that target the complement system are designed to inhibit the complement pathway at either the proximal or terminal levels. The use of a natural complement regulator such as factor H (FH) could provide a superior treatment option by restoring balance to an overactive complement system. We recently reported the moss-based production of an analog of human FH with an optimized glycan profile (CPV-104), which showed *in vitro* and *in vivo* characteristics comparable to its human counterpart. Here, we follow up our previous work, focusing in more detail on the time course and long-term efficacy of CPV-104 treatment in FH-deficient (*FH* ^–/–^) mice. The analysis of long-term treatment effects following multiple injections of human FH into mice was previously hindered by the immune response, so we developed a protocol for the sustained depletion of CD20^+^ B-cells and CD4^+^ T-cells, preventing antibody formation without influencing the C3G phenotype. Using this dual-depletion method, we were able to complete dosing interval experiments in *FH* ^–/–^ mice, administering up to three injections of CPV-104 at different intervals. Repeated CPV-104 administration was able to lastingly resolve C3 deposits, offering additional rationale for the clinical testing of CPV-104 in human C3G patients. Moreover, our novel dual-depletion method has the potential for adaptation to different mouse models, allowing the testing of multiple doses of other therapeutic proteins.

## Introduction

The complement system is a key component of innate immunity, and its major role is to protect the body against pathogens (1–3). It is controlled by a complex network of regulatory proteins that prevent excess activity and the targeting of self-cells (4). One of the key regulatory proteins is complement factor H (FH), which acts as a cofactor for factor I to cleave surface-bound C3b molecules into inactive iC3b (5, 6). FH also increases decay-accelerating activity by displacing factor Bb from C3b molecules, destabilizing the pre-formed C3 convertase. Furthermore, the C-terminal domains of FH (domains 19 and 20) bind to the host cell surface, with high specificity for sialic acid residues and heparan sulfates, thus distinguishing between self and non-self, balancing complement action, and preventing unwanted complement activation on host cells (7). Mutations or antibodies that disrupt FH function are correlated with conditions that dysregulate complement activity, such as atypical hemolytic uremic syndrome (aHUS) (8–10), C3 glomerulopathy (C3G) (11–14), and age-related macular degeneration (AMD) (15–18). Many other components are known to cause complement disbalance, leading to a broad range of complement-driven disorders (19–21).

In the last two decades, 12 complement-targeting drugs have received market approval. The first was eculizumab, approved in 2004 for the treatment of paroxysmal nocturnal hemoglobinuria (PNH). The latest are iptacopan and danicopan, approved in 2023 and 2024, respectively, also for PNH (22). Importantly, the mode of action of all these drugs is to inhibit the complement system at different levels of the cascade (22–24).

The use of a physiological regulator such as FH may provide a more sophisticated treatment option by rebalancing the complement system rather than eliciting a complete blockade. For example, treatment with FH successfully ameliorated typical C3G pathologies in *FH* ^–/–^ mice (25–27) and *FH* ^–/–^ pigs (28). However, several factors have hindered the clinical use of FH in complement-mediated disorders. Average FH serum concentrations in healthy individuals are 400–500 mg/L (29, 30), so systemic treatment would require significant amounts of purified protein. Purification of FH from donors’ blood is feasible but results in low yields (31, 32) or insufficient purity (33). High-yield recombinant FH production has been achieved in yeast, but the protein’s half-life was short due to the absence of *N*-linked glycans (34). Expression in other systems resulted in comparably low yields (35, 36). We recently reported the high-yield production of recombinant FH (CPV-104) in the moss *Physcomitrium patens* (37). CPV-104, like native serum-derived FH (sd-FH), promptly normalized serum C3 levels and rapidly degraded C3 deposits in the kidneys of *FH* ^–/–^ mice (37). The first clinical trial involving systemic treatment with recombinant FH will commence in 2025, with CPV-104 administered to C3G patients.

Here, we follow up our previous work, focusing on the time course and long-term efficacy of CPV-104 treatment in *FH* ^–/–^ mice.

Thus far, we have investigated the effect of a single dose of CPV-104 and sd-FH on C3 deposits 96 h post-injection (37). Interestingly, although the half-life of CPV-104 in mouse serum was only ~3 h, it was still detected in kidney glomeruli after 96 h and the number of C3 deposits in the kidneys of *FH* ^–/–^ mice was significantly reduced compared to controls at the same time point. This demonstrates that the pharmacodynamic (PD) effects of FH outlast the presence of the molecule in the circulation, raising questions about the duration of FH efficacy following a single injection, and the potential of repeated CPV-104 administration to prevent recurring glomerular deposits. However, analysis of long-term FH treatment in *FH^–/–^* mice is hindered by the formation of antibodies against the human protein, as also observed following the repeated administration of sd-FH (25, 26). Such antibodies caused the rapid clearance of injected FH and the deposition of IgG-FH immune complexes in the kidneys, induced anaphylaxis, and prevented repeated applications during preclinical evaluation (25).

To overcome the similar immunological response to recombinant human FH in *FH* ^–/–^ mice, we have developed a new protocol that prevents the formation of anti-FH antibodies in mice based on the continuous depletion of CD20^+^ B-cells and CD4^+^ T-cells, without influencing the C3G phenotype. This allows multiple injections of CPV-104 into *FH* ^–/–^ mice without losing functionality, leads to a lasting reduction in the number of C3 kidney deposits, and offers proof of principle for the sustained treatment of C3G with recombinant FH. This de-immunization method, which has no effect on the phenotype of animal models, opens the possibility to evaluate other recombinant therapeutic proteins that elicit antibodies in response to repeated injections.

## Materials and methods

### Complement factor H

CPV-104 was produced as described before (37). Sd-FH was purchased from Complement Technology (A137, Texas, USA).

### *In vivo* studies

*FH* ^–/–^ mice kindly provided by Matthew Pickering (Centre for Complement and Inflammation Research, Imperial College London, London, UK) were bred by Charles River Laboratories (Germany). Male and female mice 8–16 weeks of age were used for all experiments. All procedures involving animals were conducted in accordance with the guide for the care and use of laboratory animals (published by the US National Institutes of Health) and the German Animal Protection Code and were approved by local authorities (Regierungspräsidium Freiburg G-18/94, G-21/111). The mice were provided with food and water ad libitum. Mice undergoing CD4^+^ T-cell and CD20^+^ B-cell depletion were housed in individually ventilated cages to reduce the risk of infection.

Mice were injected intravenously (i.v.) with CPV-104 (40 mg/kg), sd-FH (40 mg/kg) or PBS. At the indicated time points, blood samples collected from the tail vein were used to determine serum FH and C3 concentrations. At the end of each experiment, the mice were anesthetized with an intraperitoneal (i.p.) injection of ketamine (100 mg/kg) and xylazine (15 mg/kg) and euthanized by exsanguination. Following PBS perfusion, the kidneys were harvested and either snap-frozen in liquid nitrogen or fixed in 4% paraformaldehyde (PFA). Serum samples were diluted 1:10,000 and the FH and C3 concentrations were determined as previously described (37).

### Continuous B-cell and T-cell depletion

B-cell depletion was achieved by the i.v. injection of 250 µg anti-CD20 antibody (Biolegend, USA; SA271G2). In the single-injection de-immunization protocol, the antibody was administered on days -7 and -1 prior to FH or PBS treatment. For the multiple-injection de-immunization protocol and all dose-interval studies, the antibody was administered on days -7, -1, and 7.

T-cell depletion was achieved by i.p. injection of 500 µg anti-CD4 antibody (GK1.5) (38). In the single-injection de-immunization protocol, the antibody was administered on days -4 and -1 prior to FH or PBS treatment. For the multiple-injection de-immunization protocol, the antibody was injected on days -4, -1, 2, 5, 8 and 11.

For dose-interval studies, CD4^+^ T-cell depletion was performed as follows: every 3 days, injections on days –4, –1, 2 and 5; every 4 days, injections on days –4, –1, 2, 5 and 8; and every 5 days, injections on days –4, –1, 2, 5, 8 and 11

### FACS analysis

Blood was collected in Vacutainer EDTA tubes (BD Biosciences, USA) to prevent coagulation. The Fc receptor was blocked using TruStain FcX (Biolegend; 101319) for 10 min at room temperature. B-cells were stained with the antibodies CD19-APC (eBioscience, Thermo Fisher Scientific, USA; 17-0193-82) and CD45R-APC-Cy7 (Biolegend; 103223). T-cells were stained with the antibodies CD4-FITC (Biolegend; 100510) and CD8-PerCP (Biolegend; 100732). The cell suspension was incubated for 30 min in the dark at room temperature before the erythrocytes were lysed using BD Pharm Lyse buffer (BD Biosciences; 555899) for 5 min at room temperature. After washing three times with PBS containing 2 mM EDTA, the cells were resuspended in 100 µL of the same buffer for FACS analysis using a flow cytometer (Gallios, Beckman Coulter, USA).

### FH antibody ELISA

We coated Nunc 96-well plates overnight with 5 µg/mL sd-FH (Complement Technologies, USA; A137c) at 4°C in PBS. After washing with PBS + 0.01% Tween-20 and blocking with the same buffer for 1 h at room temperature, mouse serum was diluted 1:300 and added to the plates for 1 h at room temperature. A mouse IgG1 anti-human FH antibody (AntibodyShop, UK; GAU 018-03) was used at a concentration of 62.5 ng/ml in PBS as the positive control. The plates were washed and treated with sheep anti-mouse IgG-HRP (Amersham, UK; NXA931), diluted 1:10.000, for 1 h at room temperature. The plates were washed three times and incubated with TMB substrate for 15 min, and the reaction was stopped with 2 M H_2_SO_4_. Absorption at 450 nm was measured using an Epoch plate reader (Biotek, USA).

### Immunofluorescence staining

Mouse kidneys were dissected and immediately embedded in OCT medium before freezing. We prepared 5-µm sections using a cryomicrotome. The sections were washed in PBS for 5 min before fixing in 4% PFA for 10 min. After another wash as above, the sections were blocked for 1 h in 3% bovine serum albumin in PBS. C3 was detected by staining sections with a goat anti-mouse C3 primary antibody (MPbio, USA; 0855444) overnight at 4°C, diluted 1:1,000. C3d was detected by staining the sections with a polyclonal goat anti-mouse/rat C3d antibody (R&D, USA; AF2655) overnight at 4°C, diluted 1:100. FH was detected by staining the sections with a rabbit anti-human FH antibody that recognizes short complement regulator (SCR) domains 1–4 (39) overnight at 4°C, diluted 1:100. After washing, the sections were incubated with a donkey anti-goat IgG (Thermo Fisher Scientific; A21432) diluted 1:500 or a goat anti-rabbit IgG secondary antibody (Thermo Fisher Scientific; A11034) diluted 1:1000, each for 30 min, and mounted with Vectashield containing 4′,6-diamidino-2-phenylindole (DAPI) as a nuclear counterstain. At least 35 glomeruli were analyzed per animal.

### Statistics

GraphPad Prism v8 (GraphPad Software, USA) was used for all statistical analysis and data visualization. An unpaired Student’s t-test or one-way analysis of variance (ANOVA) with Tukey’s or Dunnett’s multiple comparisons test was used to determine statistical significance as indicated (***P < 0.001, **P < 0.01, *P < 0.05).

## Results

### Efficacy time course following a single administration of CPV-104 or sd-FH to FH^–/–^ mice

**Figure 1:**
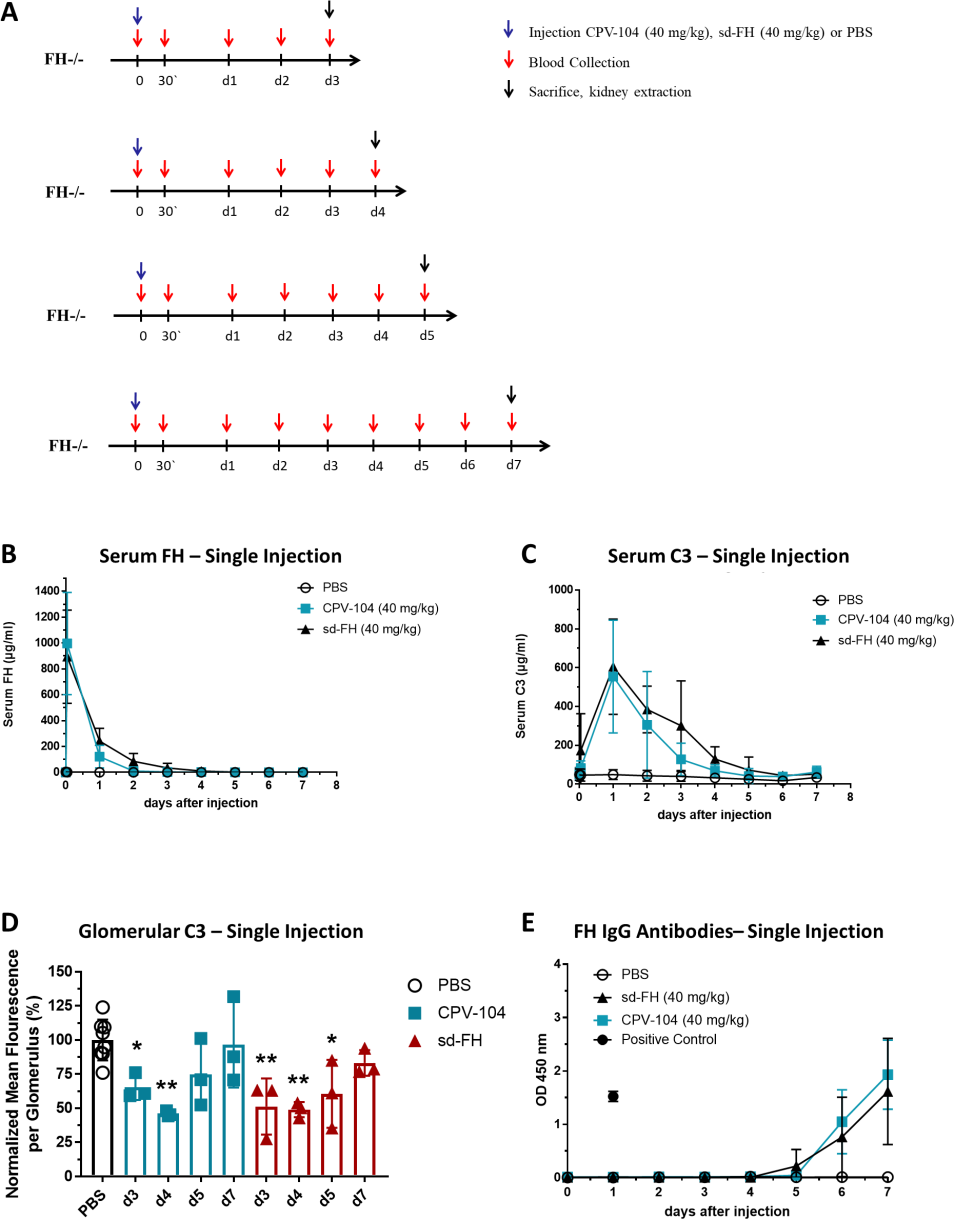
Single injections of CPV-104 and sd-FH elicit anti-FH antibodies in *FH* ^–/–^ mice. **(A)** Schematic overview of animal experiments. Mice (*n* = 2 for PBS-injected controls, *n* = 3 at each time point for mice treated with CPV-104 or sd-FH) were injected with a single dose of CPV-104, sd-FH or PBS on day 0 and blood was collected at the indicated time points. Mice were killed on day 3, 4, 5 or 7 and kidneys were harvested for histological analysis. **(B)** Serum FH levels after a single injection of CPV-104, sd-FH or PBS (data are means ± SD). **(C)** Serum C3 levels after a single injection of CPV-104, sd-FH or PBS (data are means ± SD). **(D)** Glomerular C3 deposits after a single injection of CPV-104, sd-FH or PBS. Mean fluorescence values (± SD) were normalized against PBS-treated mice on days 3, 4, 5 or 7. PBS-injected control mice are summarized as a single group. Data are means ± SD (*n* ≥ 3). Statistical significance determined by one-way ANOVA with Dunnett**’**s multiple comparisons test against PBS-treated animals (*P < 0.05; **P < 0.01). **(E)** Anti-FH IgG titers in the serum of mice after a single injection of CPV-104, sd-FH or PBS (data are means ± SD).

We injected *FH* ^–/–^ mice i.v. with a single dose of 40 mg/kg CPV-104 or sd-FH (or PBS as a control). Mice were humanely killed on days 3, 4, 5 or 7 for the analysis of blood samples and kidney tissue (**Figure 1A**). CPV-104 and sd-FH showed similar pharmacokinetic (PK) and pharmacodynamic (PD) profiles, with peak serum FH levels after ~30 min (**Figure 1B**) and peak serum C3 levels 24 h post-injection (**Figure 1C**). A single injection of CPV-104 or sd-FH reduced the number of glomerular C3 deposits, with the strongest effect observed on days 3–5 post-injection for sd-FH and days 3–4 days post-injection for CPV-104 (**Figure 1D**, **Supplementary Figure S1**). On day 7 post-injection, glomerular C3 deposits returned to levels comparable to PBS-injected mice regardless of the treatment. In contrast, FH remained present in the glomeruli for up to 7 days post-injection in both the CPV-104 and sd-FH treatment groups (**Supplementary Figure S2**). Interestingly, after just a single injection of either CPV-104 or sd-FH, FH-specific IgG was detected as early as 5 days post-injection (**Figure 1E**), with no difference in antibody response between the two treatments. These findings confirm that both CPV-104 and sd-FH trigger an immunogenic response in *FH* ^–/–^ mice, leading to the formation of specific antibodies against the human FH protein.

### Development of a continuous B-cell and T-cell dual-depletion protocol

**Figure 2:**
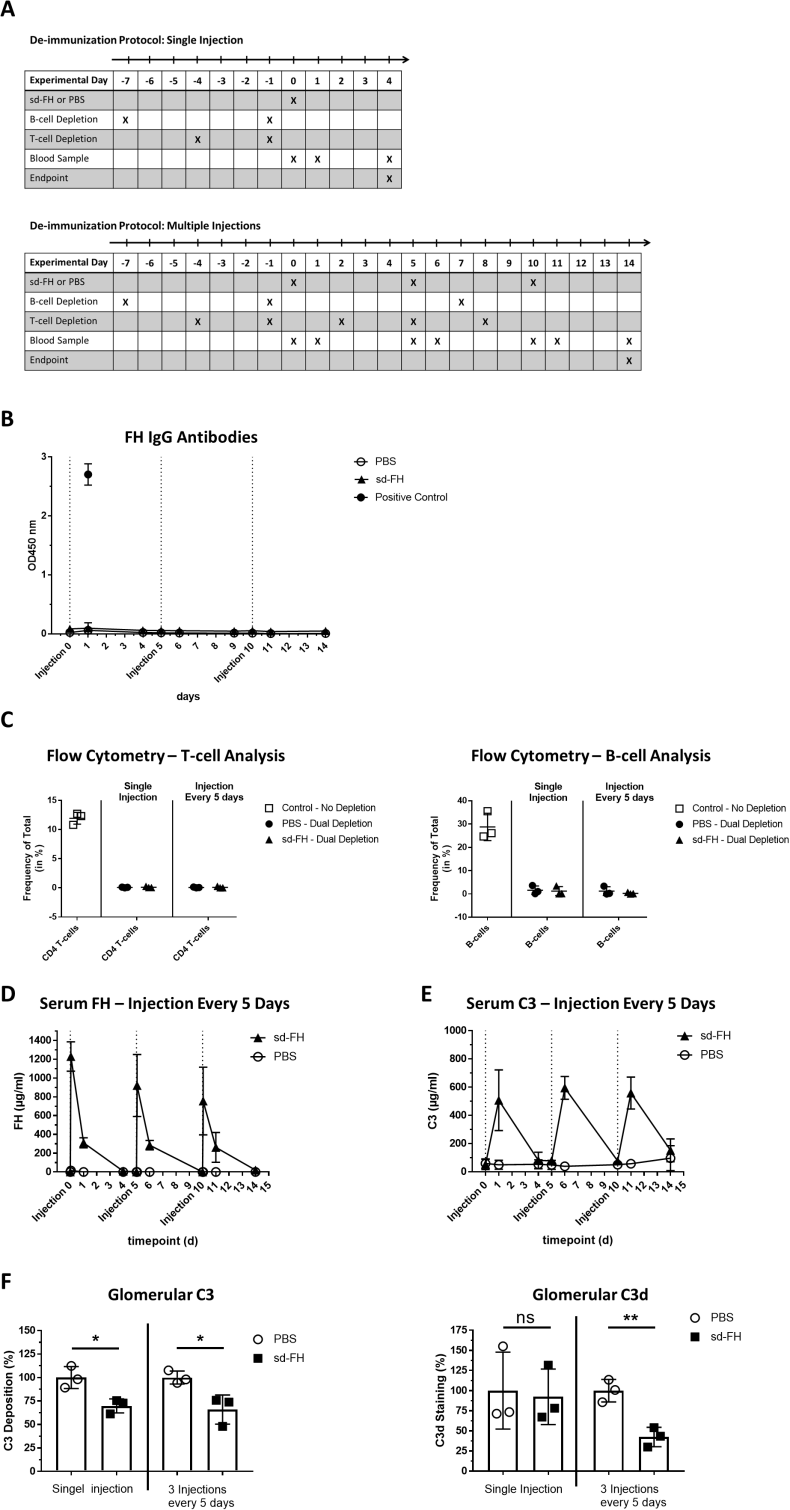
Dual-depletion protocol prevents the production of anti-FH antibodies in *FH* ^–/–^ mice. **(A)** Schematic overview of animal experiments. CD20^+^ B-cell depletion was achieved by injecting mice (i.v., *n* = 3 per group) on days –7 and day –1 with anti-CD20 antibody SA271G2 before treatment with sd-FH or PBS. For the cohort receiving three injections, B-cell depletion was repeated on day 7. CD4^+^ T-cell depletion was achieved by injecting mice (i.p., *n* = 3 per group) on days –4 and –1 with anti-CD4 antibody GK1.5 before treatment with sd-FH or PBS. For the cohort receiving three injections, T-cell depletion was repeated on days 2, 5, 8 and 11. Treatments with sd-FH or PBS were carried out on days 0, 5 and 10 and blood was collected at the indicated time points. Mice were killed on days 4 or 14 and kidneys were harvested for histological analysis. **(B)** Anti-FH IgG titers in the serum of mice subjected to the dual-depletion protocol followed by three injections of sd-FH or PBS at 5-day intervals (data are means ± SD, *n* ≥ 3). **(C)** FACS analysis of CD4^+^ T-cells (left) and CD19^+^ CD45^+^ B-cells (right) in *FH* ^–/–^ mice subjected to the dual-depletion protocol, showing almost complete depletion by day 14. **(D)** Serum FH levels in the cohorts of mice described in panel **(A)** (data are means ± SD, *n* = 3). **(E)** Serum C3 levels in the cohorts of mice described in panel **(A)** (data are means ± SD, *n* = 3). **(F)** Analysis of glomerular C3 and C3d deposits in the depleted mice described in panel **(A)**. Mean fluorescence values (± SD) were normalized against PBS-treated mice. Statistical significance was determined using Student**’**s t-test (*P < 0.05; **P < 0.01; ns, not significant).

To prevent the formation of anti-FH antibodies, we evaluated a protocol for the depletion of CD20^+^ B-cells in *FH* ^–/–^ mice using an anti-CD20 antibody. However, B-cell depletion alone was not sufficient to prevent antibody formation (**Supplementary Figure S3**). We therefore adapted the protocol by including an anti-CD4 antibody to simultaneously deplete CD4^+^ T-cells. Mice were injected with both antibodies before the administration of sd-FH as a single dose or three doses spaced 5 days apart, with PBS as a control (**Figure 2A**). Blood samples and kidney tissues were collected from mice killed 4 days after the single injection or 4 days after the third injection, respectively.

Whereas FH-specific IgG titers increased in the mice excluded from the dual-depletion protocol, we did not detect such antibodies in the depleted cohorts even on day 14 in the multiple-dose group, 4 days after the third injection (**Figure 2B**). FACS analysis confirmed the efficient (>95%) depletion of CD20^+^ B-cells and CD4^+^ T-cells in all tested mice (**Figure 2C**, **Supplementary Figures S4A, B**). Peak FH and C3 serum concentrations were comparable between the first and third injections (**Figures 2D, E**) and the number of kidney C3 deposits was lower, similar to the results observed 4 days after the first injection (**Figure 2F**, **Supplementary Figure S5**). Interestingly, whereas a single administration of sd-FH did not significantly reduce the number of C3d deposits, three consecutive injections led to a significant decrease (**Figure 2F**, **Supplementary Figure S5**). To assess whether depletion affected the C3G phenotype, periodic acid-Schiff (PAS) staining was applied to kidney sections (**Supplementary Figure S6**). *FH* ^–/–^ mice have slightly accentuated basement membranes without definite signs of membranoproliferative cellular interposition compared to wild-type mice. More importantly, no histological differences could be detected between depleted and non-depleted *FH* ^–/–^ mice.

### The dual-depletion protocol facilitates the assessment of CPV-104 dosing regimens

**Figure 3:**
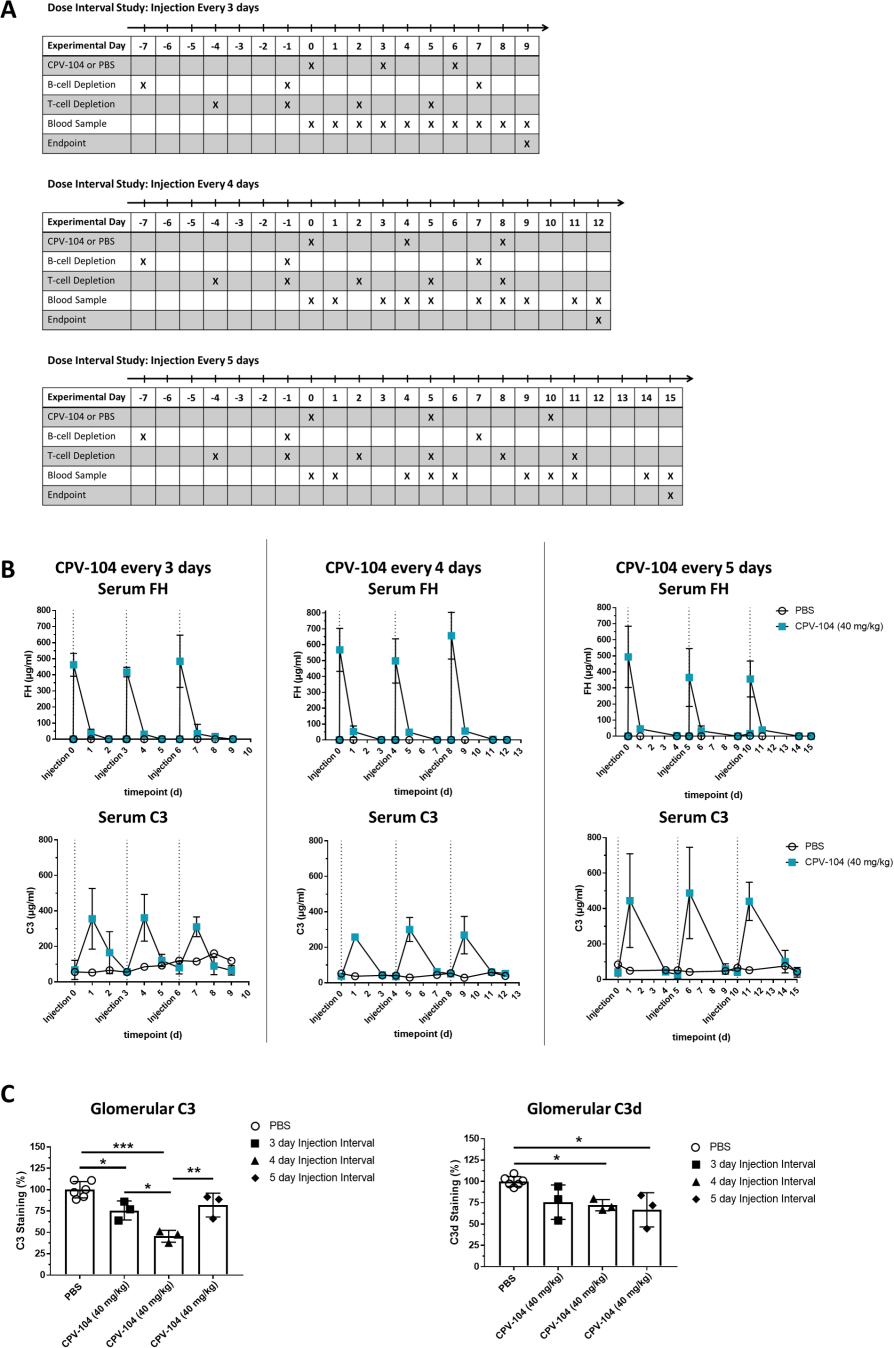
The dual-depletion protocol facilitates the testing of CPV-104 dosing regimens in *FH* ^–/–^ mice. **(A)** Schematic overview of animal experiments. The mice (*n* = 2 for PBS injected controls, *n* = 3 at each time point for mice treated with CPV-104) were injected three times with either CPV-104 or PBS at dosing intervals of 3, 4 or 5 days. Blood samples were collected as indicated, and mice were killed 3, 4 or 5 days after the last injection and kidneys were harvested for histological analysis. **(B)** Serum FH and C3 concentrations in dual-depleted FH^–/–^ mice (*n* = 2 for PBS injected controls, *n* = 3 at each time point for mice treated with CPV-104) receiving three injections of CPV-104 or PBS every 3, 4 or 5 days, and the status of glomerular deposits. Blood samples were collected as indicated to monitor the serum concentrations of FH (top panels) and C3 (bottom panels), both of which remained consistent after each injection (data are means ± SD). **(C)** CPV-104 significantly reduced the number of glomerular C3 deposits with the strongest effect observed using a 4-day dosing interval. The number of glomerular C3d deposits was also significantly reduced when using 4-day or 5-day dosing intervals. PBS-injected control mice are summarized as a single group. Data are means ± SD (*n* ≥ 3). Statistical significance was determined by one-way ANOVA with Tukey**’**s multiple comparisons test (*P < 0.05; **P < 0.01; ***P < 0.001).

Having confirmed that the dual-depletion protocol prevents the formation of FH-specific antibodies in *FH* ^–/–^ mice injected with sd-FH, we next attempted to establish an optimal dosing interval for CPV-104, aiming for the permanent elimination of kidney deposits and normalization of the C3 level. Accordingly, we injected CPV-104 (or PBS as a control) into dual-depleted *FH* ^–/–^ mice with an initial schedule of three doses at intervals of 3, 4 or 5 days (**Figure 3A**). Blood was collected at the indicated time points and the mice were killed humanely for the extraction of kidneys on days 9, 12 or 15 after the first CPV-104 injection (**Figure 3A**).

As before, FACS analysis confirmed the efficient depletion of CD20^+^ B-cells and CD4^+^ T-cells (**Supplementary Figure S7**), and no FH-specific IgG was detected by ELISA (**Supplementary Figure S8**). In accordance with previous results, PK analysis revealed peak FH concentrations ~30 min after each administration, resulting in comparable C_max_ levels of CPV-104 in serum after each injection (**Figure 3B**, upper panel). C3 levels in serum reached peak concentrations 24 h post-injection, as observed in previous experiments (**Figure 3B**, lower panel), regardless of the dosing schedule. Although the C3 level in serum was not completely normalized by one of the three dosing regimens, glomerular C3 and C3d deposits decreased in all cases (**Figure 3C**, **Supplementary Figure S9**), with the lowest fluorescence intensity for C3 deposits achieved with dosing every 4 days. Glomerular staining for FH indicated a more prolonged effect on the kidney compared to C3 concentrations alone (**Supplementary Figure S9**). CPV-104 was still present and detectable in the kidneys of all injected mice, independent of the dosing interval.

## Discussion

Recombinant proteins are widely used as therapeutics, particularly monoclonal antibodies for the treatment of cancers and autoimmune disorders, and recombinant human proteins as replacement therapy in genetic disorders caused by the loss or dysfunction of native proteins (40, 41). In preclinical proof-of-concept studies in animal models, a common challenge is the immune response triggered by human proteins, particularly during long-term treatment evaluations. The elicitation of antibodies against therapeutic human proteins has previously been reported following the repeated injection of recombinant human FH into mice (25, 26). Although the efficacy of sd-FH in *FH^–/–^* mice was confirmed, assessment of the beneficial effects of repeated injections was hindered by the antibodies and resulting immune complexes, which accumulated in the kidneys. Even recombinant murine FH triggered antibody formation, which was attributed to differences in glycan structures between the recombinant protein and native murine FH (26).

Preclinical testing in animal models delivers insights into the PK/PD characteristics of therapeutic proteins, so strategies that suppress antibody formation, without influencing the model’s phenotype, are of great value. We confirmed that *FH* ^–/–^ mice injected with human sd-FH or the glycan-optimized FH analog produced in moss (CPV-104) respond by producing high titers of anti-FH IgG, starting as early as 5 days after the first injection (**Figure 1E**). Because antibodies are produced by CD20^+^ B-cells, we initially tested the depletion of these cells by injecting anti-CD20 antibodies 3 days before treatment with sd-FH. This was insufficient, so we added a second antibody to deplete CD4^+^ T-cells, which amplify the activity of B-cells, leading to stronger and longer-lasting antibody responses (42). The predominance of IgG-type anti-FH antibodies in our preliminary experiments indicated that the immune response to sd-FH was T-cell dependent, supporting a dual-depletion approach (43). Our method involved the injection of commercially available anti-CD20 and anti-CD4 antibodies into *FH* ^–/–^ mice followed by treatment with sd-FH either as a single dose or as three doses at 5-day intervals. The simultaneous depletion of CD20^+^ B-cells and CD4^+^ T-cells was successful, as confirmed by FACS analysis using corresponding detection antibodies (**Figure 2C**), and although the titer of anti-FH antibodies increased after 5 days in non-depleted mice, we detected no anti-FH antibodies in mice subjected to the dual-depletion protocol (**Figure 2B**). There were no discernable negative effects on the mice and the *FH* ^–/–^ phenotype remained identical to that of the non-depleted *FH* ^–/–^ cohort (**Supplementary Figure S6**). In agreement with previous results, serum FH levels peaked after ~30 min and C3 levels 24 h after the injection of sd-FH in both cohorts. The depleted cohort responded similarly to the first and third FH injections in terms of peak FH levels and changes in serum C3 levels, confirming the absence of a deleterious effect caused by anti-FH-antibodies.

We used the new protocol to establish a dosing regimen for our glycan-optimized FH analog CPV-104 (37), aiming to permanently eliminate pathogenic kidney C3 fragment deposits and normalize the C3 serum level. Although the simultaneous depletion of CD20^+^ B-cells and CD4^+^ T-cells prevented the formation of anti-FH antibodies (**Supplementary Figures S7**, **S8**) none of the three tested dosing intervals achieved steady-state serum FH or C3 concentrations (**Figure 3B**). However, all three dosing intervals led to a significant reduction in the number of glomerular C3 and C3d deposits, confirming the long-term efficacy of CPV-104. A significant reduction in the number of C3d deposits was observed only following repeated injections of either sd-FH or CPV-104 (**Figure 3C**, **Supplementary Figures S5**, **S9**), consistent with previous findings (25). This significant reduction of C3d was only seen after 5 or 10 daily injections of sd-FH. However, after 10 daily injections, anti-FH antibodies triggered albuminuria, uremia, and signs of proliferative glomerulonephritis in *FH* ^–/–^ mice (25). Despite the presence of these antibodies, sd-FH was still detected in the glomeruli, and C3d deposits were still significantly reduced. In clinical practice, C3d is a marker of ongoing complement activation and C3 consumption (44). Although eculizumab treatment reduces plasma C3d levels in aHUS patients, C3d levels increase in C3G patients, indicating that complement inhibition at the C3 level could be more beneficial for the treatment of C3G (45).

The findings described above support our observation that, although the restricted half-life of CPV-104 and sd-FH in circulation may limit their long-term ability to prevent complement hyper-activation in the fluid phase, their PD effects in the kidneys are more sustained. Indeed, CPV-104 and sd-FH can be detected up to 7 days post-injection in the glomeruli (**Supplementary Figure S2**), suggesting that FH either binds to glomerular endothelial cells or their glycocalyx (46) or binds to/interacts with C3 deposits directly and remains *in situ* longer than in circulation. Similar results have been observed in FH deficient mice treated with ADX-097, a fusion protein combining a humanized anti-C3d monoclonal antibody with the first five short consensus repeats of FH (47). The authors demonstrate that tissue complement inhibition could be achieved at sites of complement activation while avoiding complete blockade of complement in circulation.

Even validated models such as *FH* ^–/–^ mice are not entirely consistent with C3G pathology in humans, given that the complete loss of FH is rare in human patients, and that autoantibodies against C3Nef (absent in *FH* ^–/–^ mice) play a key role in C3G pathogenesis (48, 49). However, the fast and long-lasting positive effect of CPV-104, reducing the number of C3 deposits in the kidneys (which is not strictly correlated with the half-life in circulation), and the potential of repeated administrations to lastingly resolve the C3 and especially C3d deposits, are important *in vivo* observations, providing additional rationale for the clinical testing of CPV-104 in C3G patients.

More generally, our dual-depletion protocol (used here to study the efficacy of repeated CPV-104 administration in mice) has the potential to add value to other preclinical studies by allowing the analysis of multiple dosing regimens in the absence of confounding antibody responses, such as ADX-097, MFHR1, MFHR13 or homodimeric minimal FH (47, 50–52). However, pilot experiments are needed to assess the suitability of the protocol for other diseases and therapeutic proteins because the nature of the antibody response differs between models, reflecting the degree of divergence between human and mouse orthologs of different therapeutic proteins and the conservation of glycan structures in recombinant proteins.

## Data Availability

The raw data supporting the conclusions of this article will be made available by the authors, without undue reservation.
