# Supplementary material for: Effective long-term treatment with moss-produced factor H by overcoming the antibody response in a mouse model of C3G
Source: Front Immunol. 2025 Mar 7;16:1535547. doi: 10.3389/fimmu.2025.1535547 (PMC11925764; doi:10.3389/fimmu.2025.1535547)
Supplement: Supplementary file 1 [file DataSheet1.docx]

Supplementary Material

# Supplementary Figures

## Supplementary Figure 1


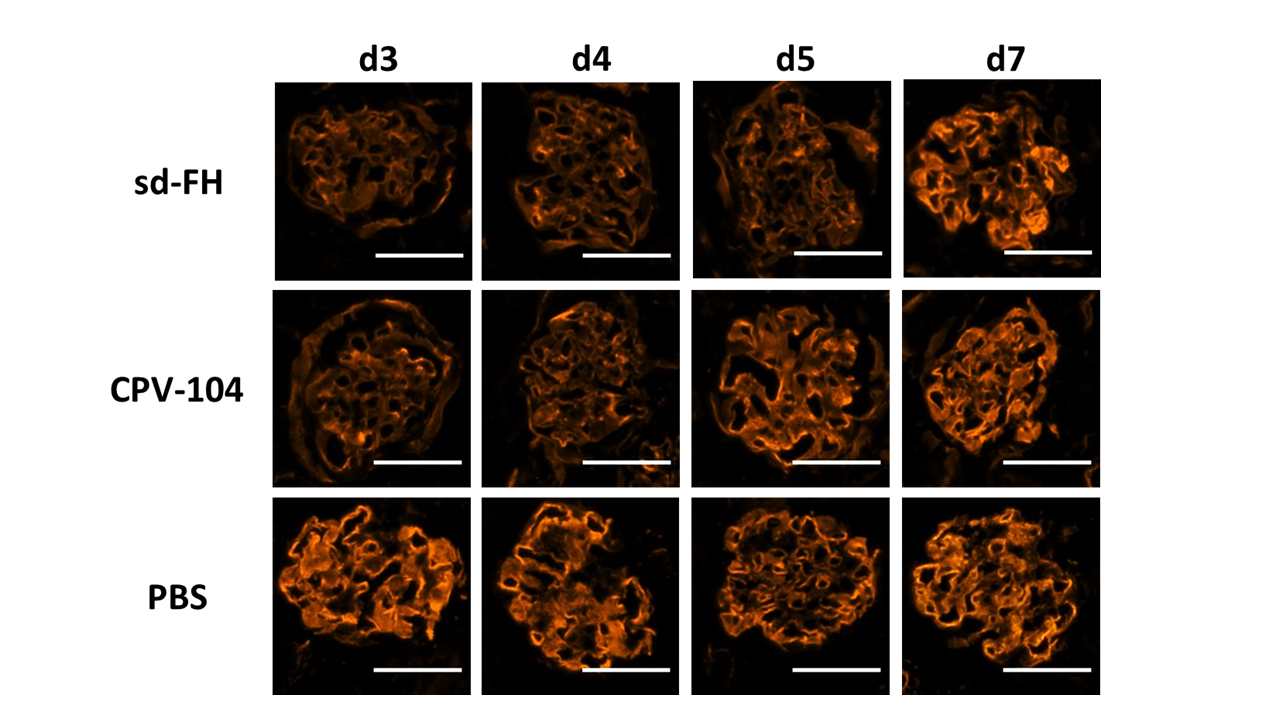


**Figure S1.** Representative images showing the time course of glomerular C3 deposits in *FH*^–/–^ mice injected with CPV-104, sd-FH or PBS. Mice received a single i.v. injection of CPV-104 (40 mg/kg), sd-FH (40 mg/kg) or PBS, and were killed 3, 4, 5 or 7 days post-injection as depicted in figure 1A (*n* = 3 per time point). In the CPV-104 treatment group, glomerular C3 deposits began to return ~5 days post-injection, whereas fewer deposits were observed in sd-FH treated mice up to day 5. By day 7, C3 deposits had returned to PBS levels, regardless of treatment. Scale bar = 40 µm.

## Supplementary Figure 2


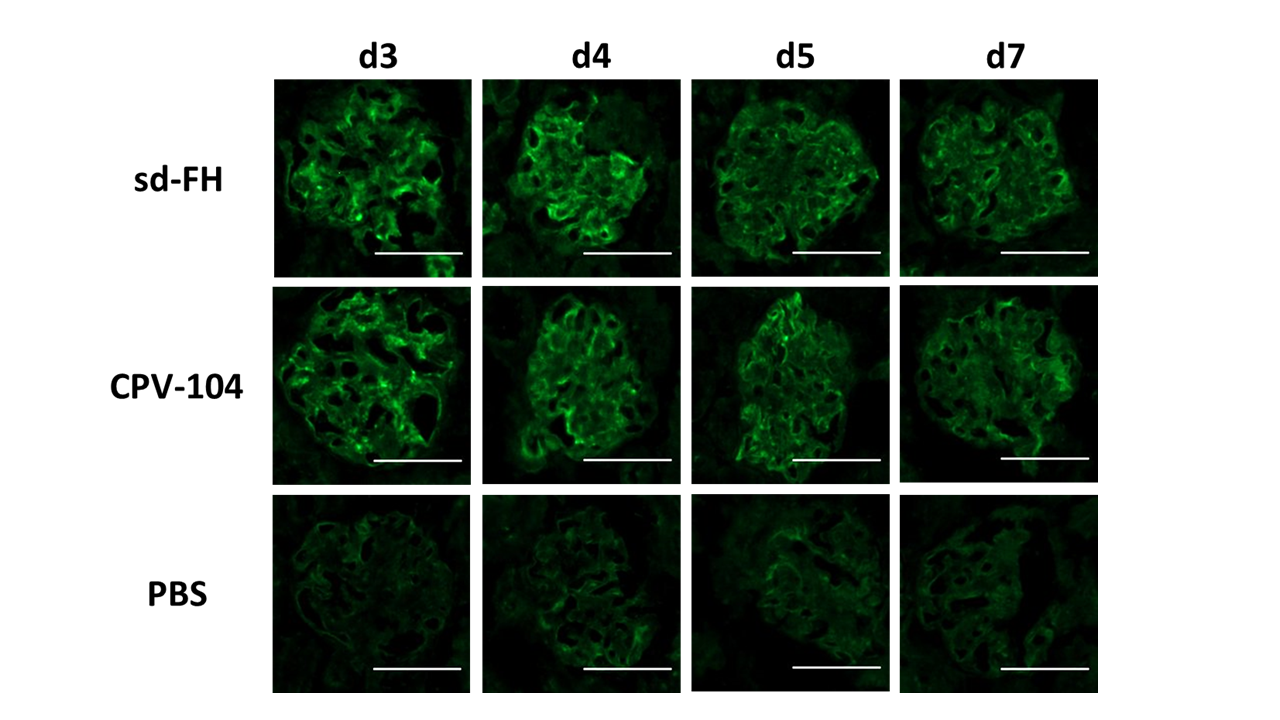


**Figure S2**: Representative images showing FH in *FH*^–/–^ mice injected with CPV-104, sd-FH or PBS. Mice received a single i.v. injection of CPV-104 (40 mg/kg), sd-FH (40 mg/kg) or PBS, and were killed 3, 4, 5 or 7 days post-injection as depicted in figure 1A. FH was detected in the kidneys of mice in the CPV-104 and sd-FH treatment groups up to 7 days post-injection. Scale bar = 40 µm.

## Supplementary Figure 3


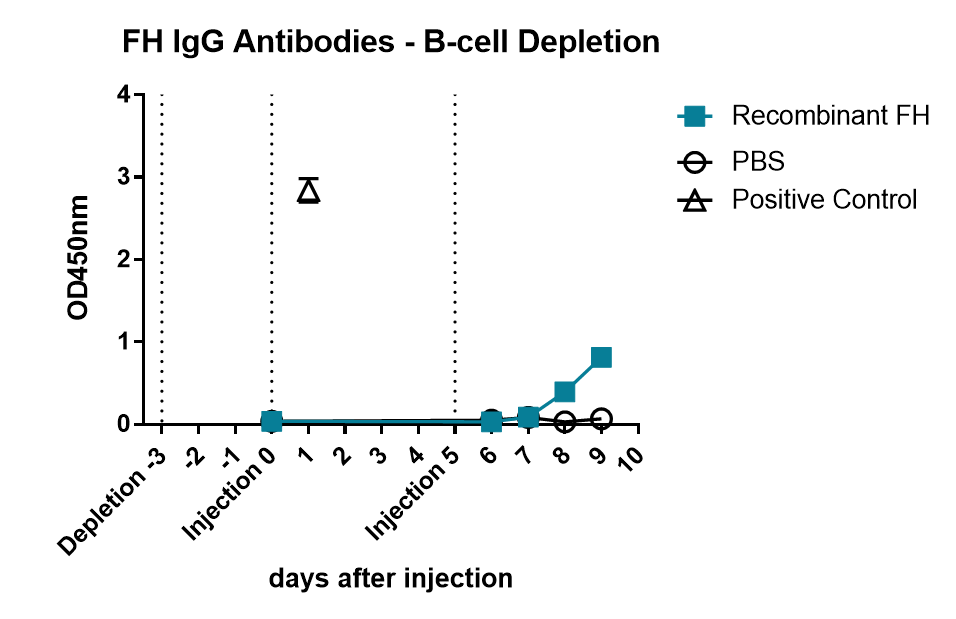


**Figure S3:** B-cell depletion does not prevent the antibody response against FH in *FH*^–/–^ mice. CD20^+^ B-cells were depleted by injecting mice once i.p. with 250 µg of an anti-CD20 antibody 3 days before the injection of either CPV-101 (40 mg/kg) or PBS on days 0 and 5. Blood was collected at the indicated time points and the mice were killed on day 9. Mice injected with recombinant FH developed antibodies by day 8 following the second injection.

## Supplementary Figure 4


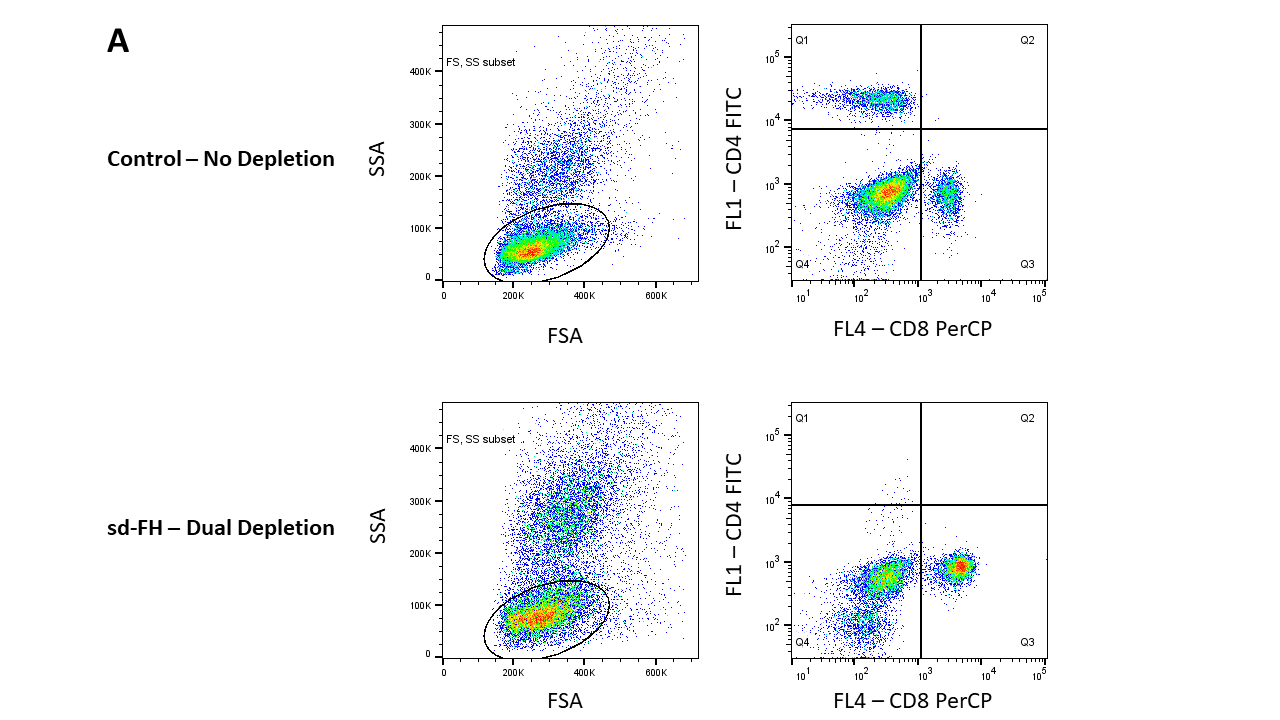


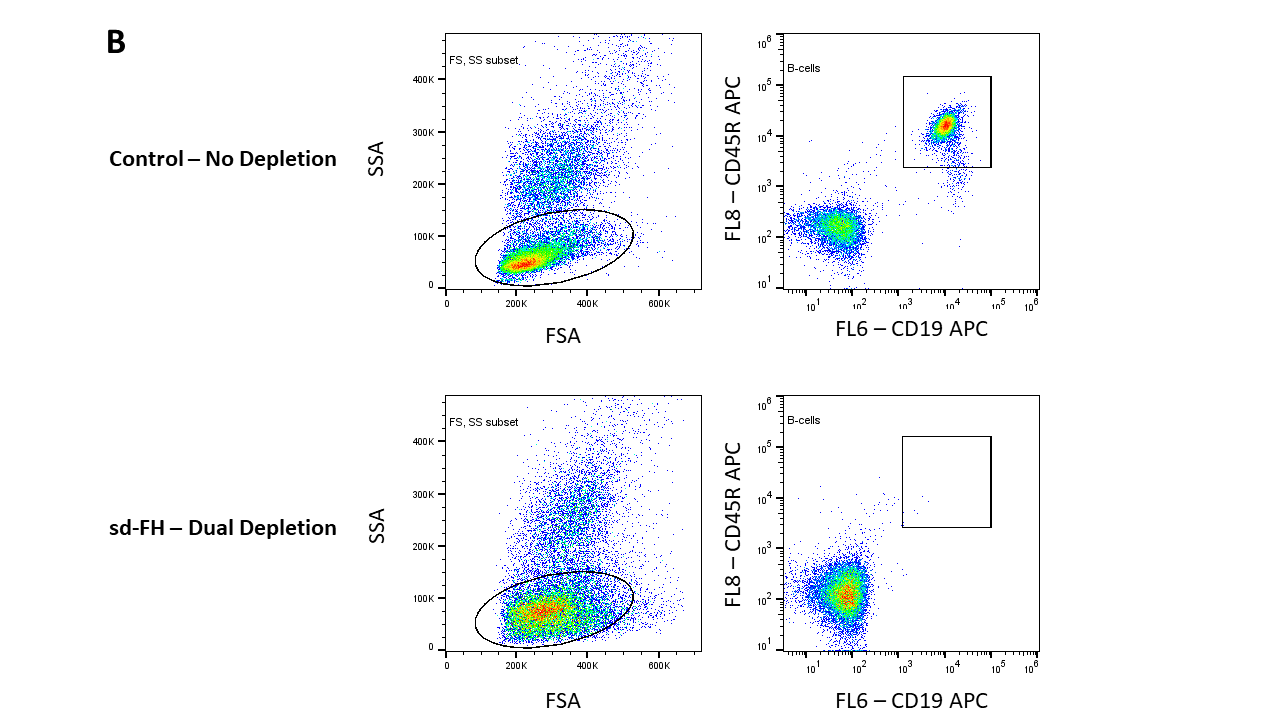


**Figure S4:** Representative images of the FACS gating strategy for the detection of B-cells and CD4^+^ T-cells in the blood of untreated and dual-depleted *FH*^–/–^ mice. CD4^+^ T-cell and CD20^+^ B-cell depletion was performed as depicted in figure 2A (De-immunization Protocol: Multiple Injections). **(A)** Whole blood was collected from the mice and stained with anti-CD4 and anti-CD8 antibodies. The top panel shows the normal distribution of CD4^+^ and CD8^+^ T-cells in the blood of untreated *FH*^–/–^ mice. The bottom panel shows the almost complete absence of CD4^+^ T-cells in mice subjected to the dual-depletion protocol. **(B)** Whole blood was collected from the mice and stained with anti-CD19 and anti-CD45R antibodies to detect B-cells. The top panel shows the normal distribution of CD19^+^ CD45R^+^ B-cells in the blood of untreated *FH*^–/–^ mice. The bottom panel shows the almost complete absence of CD19^+^ CD45R^+^ B-cells in mice subjected to the dual-depletion protocol.

## Supplementary Figure 5


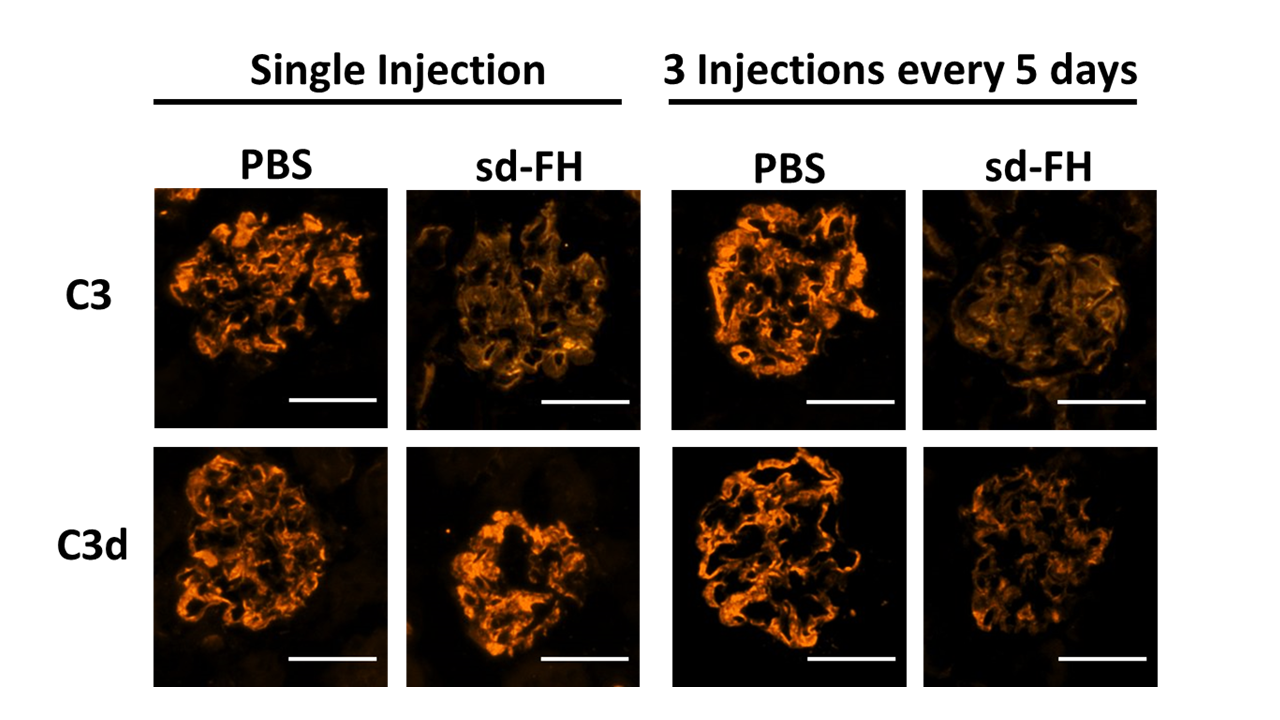


**Figure S5**: Representative images showing glomerular C3 and C3d deposits in *FH*^–/–^ mice subjected to the dual-depletion protocol before injection with sd-FH as depicted in figure 2A. Mice received either a single injection or three injections at 5-day intervals. Mice were killed 4 days after the first or third injection, and kidneys were harvested for analysis. Although the reduction in the number of glomerular C3 deposits was similar between one and three injections, only multiple injections of FH led to a reduction in the number of glomerular C3d deposits. Scale bar = 40 µm.

## Supplementary Figure 6


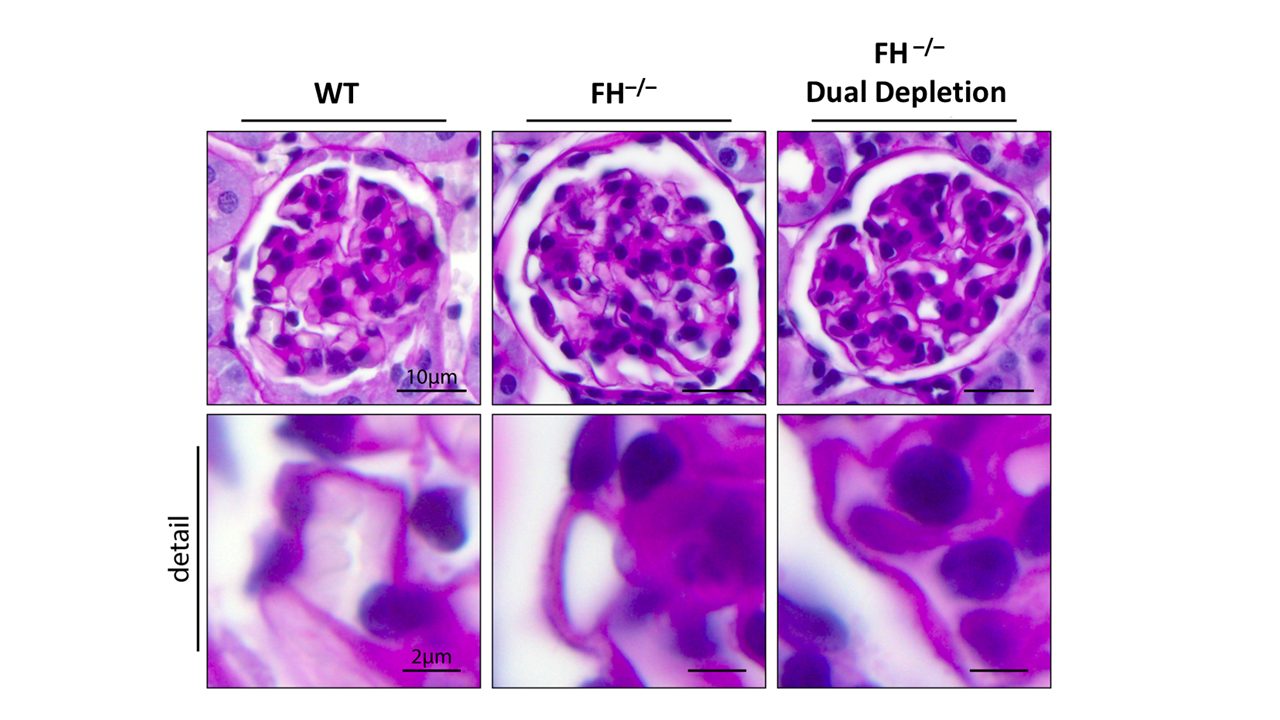


**Figure S6:** The depletion of CD4^+^ T-cells and CD20^+^ B-cells does not alter the C3G phenotype in *FH^–/–^* mice. Representative images are shown of PAS-stained kidneys from untreated wild-type and *FH^–/–^* mice, as well as FH^–/–^ mice which underwent dual depletion. CD4^+^ T-cell and CD20^+^ B-cell depletion was performed as depicted in figure 2A (De-immunization Protocol: Multiple Injections). Histological evaluations revealed no changes in glomerular morphology between depleted and untreated FH^–/–^ mice.

## Supplementary Figure 7


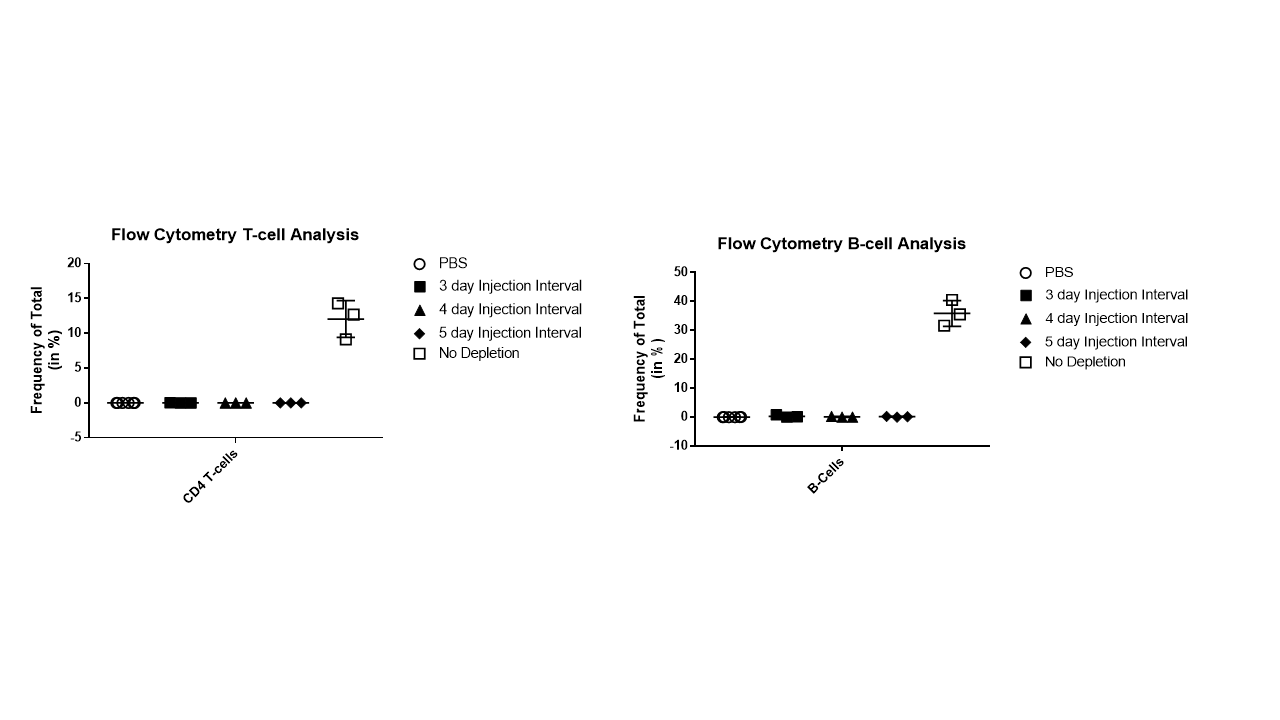
**Figure S7**: Flow cytometry analysis of whole blood CD4^+^ T-cells (left) and B-cells (right) from *FH^–/–^* mice in the dose interval study at the end of the experiment as depicted in figure 3A. CD4^+^ T-cells as well as CD19^+^ CD45R^+^ B-cells were efficiently depleted until the end of the experiment. Data are means ± SD (n ≥ 3).

## Supplementary Figure 8


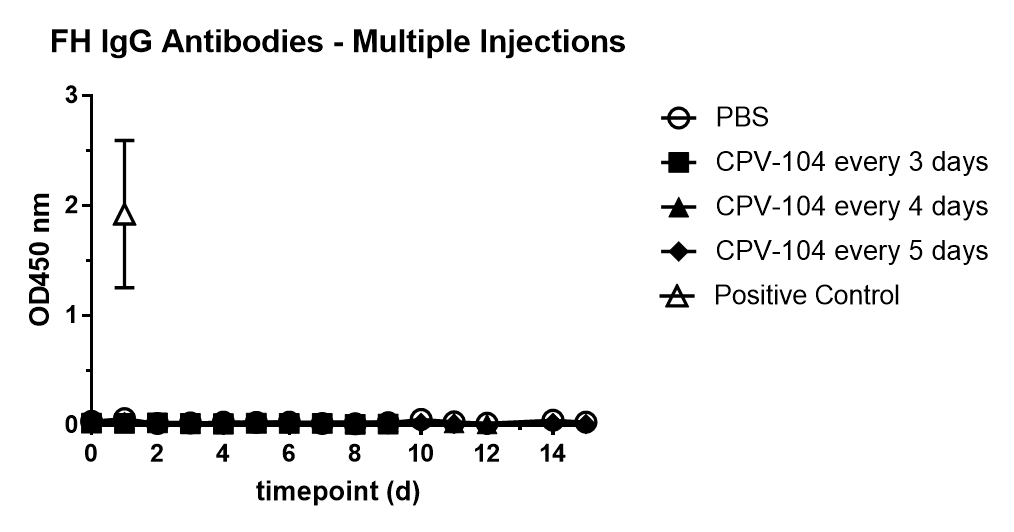


**Figure S8:** *FH*^–/–^ mice did not develop anti-FH antibodies during the dosing interval study with CPV-104 as depicted in figure 3A. After depleting *FH*^–/–^ mice, CPV-104 was administered three times at intervals of 3, 4 or 5 days. Blood samples were collected at the indicated time points and no anti-FH antibodies were detected in any of the treated animals. Data are means ± SD (*n* = 3).

## Supplementary Figure 9


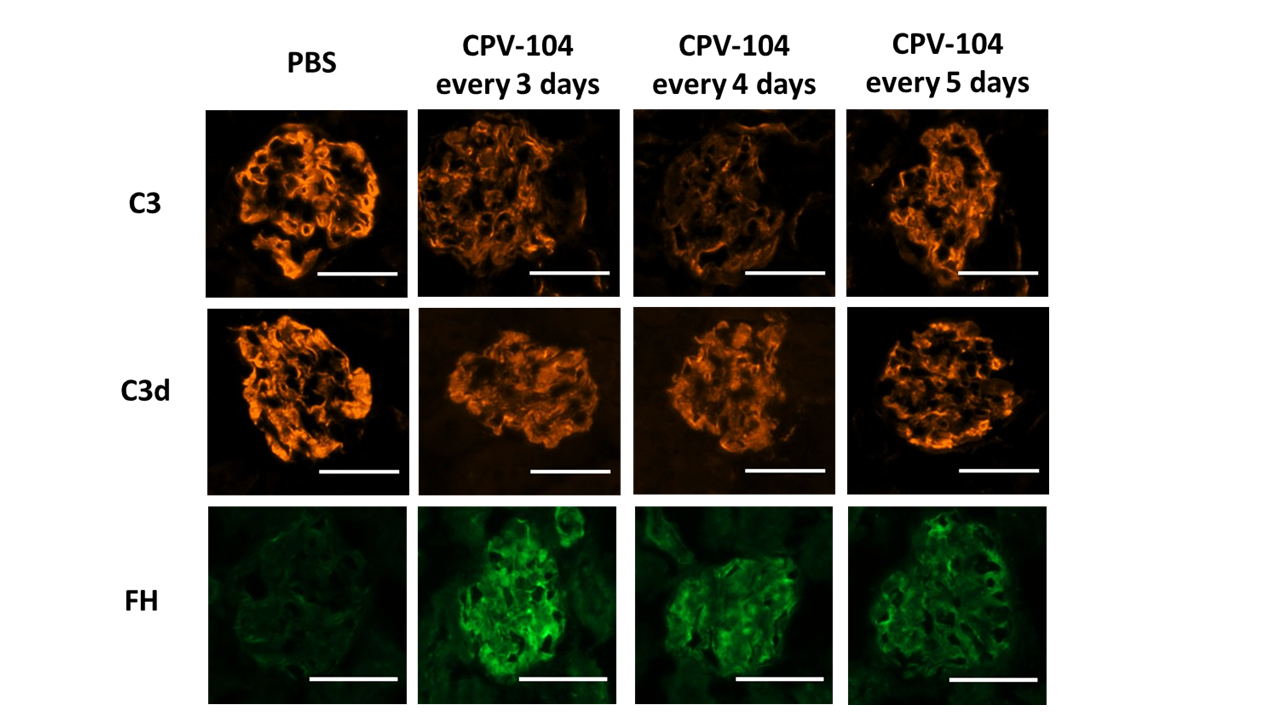


**Figure S9:** Multiple injections of CPV-104 result in the long-term dissolution of glomerular C3 deposits. Representative images of glomerular C3, C3d and FH staining are shown in dual-depleted *FH*^–/–^ mice receiving three injections of CPV-104 or PBS every 3, 4 or 5 days as depicted in figure 3A. Scale bar = 40 µm.
